# The United Confederate Veterans

**Published:** 1893-08

**Authors:** 


### THE UNITED CONFEDERATE VETERANS.

We have received from Dr. Joseph Jones, of New Orleans, Surgeon-General of the United Confederate Veterans, his fourth official report, for the period extending from April, 1892, to July, 1893. The number of camps now organized is two hundred and fifty-one. The entire South should be covered by camps of Confederate Veterans, and the organization of each should be placed in permanent form by publication. The objects of this Association are:

1. The preservation of the story of our heroic struggle, with its victories, defeats, disasters, privations and sufferings.

2. The relief of the sufferings, diseases and wounds of the veterans of the Confederate army and navy.

These objects are only to be accomplished by organization and generous co-operation. Since the formation of this union of Confederate veterans, Commodore Hunter, General Beauregard, General Kirby Smith and President Davis, with a host of brave officers and soldiers, have answered the last call. Dr. Jones has addressed a circular to the commander of each camp, calling for the number of members, the officers in command, the number of disabled soldiers in the same, the number of deaths since organization, the number of widows of soldiers aided by the camp, the amount of money annually appropriated for the support of soldiers and the relief of widows, and other information. Up to the 10th of April, 1893, only one hundred of the two hundred and fifty-one camps replied to the above circulars. These one hundred represented a little less than ten thousand Confederate veterans. In these one hundred camps there are only two hundred and seventy disabled soldiers.

The number of deaths reported in these camps since this formation has been four hundred and seventy-one. The disabled and indigent soldiers, and the indigent widows of soldiers supported by the camps have amounted to an insignificant number. These statistics indicate the independence and substantial thrift and prosperity of the Confederate veterans throughout the South. Camps wishing to communicate with the Surgeon-General on this matter should address Dr. Joseph Jones, 156 Washington avenue, New Orleans, La.

